# Impact of free hypertension pharmacy program and social distancing policy on stroke: A longitudinal study

**DOI:** 10.3389/fpubh.2023.1142299

**Published:** 2023-04-18

**Authors:** Qi Zhou, Meihua Yu, Meihua Jin, Peng Zhang, Guoyou Qin, Ye Yao

**Affiliations:** ^1^Department of Biostatistics, School of Public Health and The Key Laboratory of Public Health Safety of Ministry of Education, Fudan University, Shanghai, China; ^2^Huzhou Center for Disease Control and Prevention, Huzhou, Zhejiang, China

**Keywords:** stroke, hypertension, pharmaceutical intervention program, social distancing, Serfling regression model

## Abstract

**Background:**

The estimated lifetime risk of stroke was the highest in East Asia worldwide, especially in China. Antihypertensive therapy can significantly reduce stroke mortality. However, blood pressure control is poor. Medication adherence is a barrier as patients’ out-of-pocket costs have risen. We aimed to take advantage of a free hypertension pharmacy intervention and quantified the impact on stroke mortality.

**Methods:**

A free pharmaceutical intervention program was implemented in Deqing, Zhejiang province in April 2018. Another non-pharmaceutical intervention, social distancing due to the pandemic of Coronavirus disease 2019 (COVID-19), was also key to affecting stroke mortality. We retrospectively collected the routine surveillance data of stroke deaths from Huzhou Municipal Center for Disease Prevention and Control in 2013–2020 and obtained within-city mobility data from Baidu Migration in 2019–2020, then we quantified the effects of both pharmaceutical intervention and social distancing using Serfling regression model.

**Results:**

Compared to the predicted number, the actual number of stroke deaths was significantly lower by 10% (95% CI, 6–15%; *p* < 0.001) from April 2018 to December 2020 in Deqing. Specifically, there was a reduction of 19% (95% CI, 10–28%; *p* < 0.001) in 2018. Moreover, we observed a 5% (95% CI, −4 – 14%; *p* = 0.28) increase in stroke mortality due to the adverse effect of COVID-19 but it wasn’t statistically significant.

**Conclusion:**

Free hypertension pharmacy program has great potential to prevent considerable stroke deaths. In the future, the free supply of low-cost, essential medications that target patients with hypertension at increased risk of stroke could be taken into account in formulating public health policies and guiding allocations of health care resources.

## Introduction

Stroke remains the leading cause of mortality and morbidity worldwide (1). The estimated lifetime risk of stroke was the highest in East Asia, with China facing the greatest challenges especially (2, 3). The risk factors for stroke are high systolic blood pressure, smoking, diabetes, and lack of physical activity (3, 4). High systolic blood pressure accounted for 2.54 million deaths in 2017 in China, of which 95.7% were due to cardiovascular diseases (CVD) (3). Growing evidence suggests pharmacological blood pressure lowering for primary and secondary prevention of CVD across different levels of blood pressure is cost-effective (5, 6). Earlier clinical trials in Chinese Older Adult people confirmed that active systolic antihypertensive therapy can significantly reduce the mortality and morbidity of stroke in hypertensive patients (7, 8).

Prescription medications have experienced many years of growth in costs, and as deductibles and co-pays have risen, so have patient out-of-pocket costs (9). It is hypothesized that higher costs can lead to potentially harmful changes in patient behavior, including taking lower doses than recommended or discontinuing beneficial medications (10). If medication adherence is a barrier, blood pressure will not be significantly reduced, nor will downstream cerebrovascular health (11, 12). The 2018 China Chronic Disease and Risk Factor Surveillance (CCDRFS) study showed that only one-third of Chinese hypertensive patients received antihypertensive treatment, and of those receiving treatment, 11% had blood pressure effectively controlled (13). While results of cross-sectional analyses from the National Health and Nutrition Examination Survey (NHANES) showed that the hypertension treatment was higher than 50% among US adults aged ≥25 and among those treated, over 60% had blood pressure controlled well (14). The prevention and control of non-communicable diseases in China are not optimistic compared with other countries and it has caused a major economic burden (14, 15). However, few studies have focused on the effect of low-cost essential medications provided for free by the local government on stroke. It is unclear, to what extent the implementation of a free pharmacy program could decrease the risk of stroke mortality.

Social distancing decreases an individual’s likelihood of contracting Coronavirus disease 2019 (COVID-19), however, the risk of chronic diseases due to lack of physical activities and social support is increasing. As recommended, all adults, even those with chronic conditions, should engage in at least 150 min a week of moderate-intensity exercise (16). However, in early 2020 due to the pandemic of COVID-19, in which nationwide social distancing was implemented, the intensity of exercise greatly decreased and the time of stay-at-home increased.

In this study, we aimed to take advantage of the free hypertension pharmacy program in Deqing County, Huzhou, Zhejiang Province to investigate the effectiveness and degree of pharmaceutical intervention in decreasing the disease burden of stroke. We quantified the impact of pharmaceutical intervention on stroke mortality. In addition, we assessed the changes in stroke mortality due to the pandemic of COVID-19, to disentangle the social distancing effect from the pharmaceutical intervention effect.

## Methods

### Pharmaceutical intervention program in Deqing

Huzhou, a northern city in Zhejiang Province, consists of two districts Wuxing, Nanxun, and three counties Deqing, Anji, and Changxing. From April 1, 2018, Deqing County began launching a free pharmaceutical intervention program, designed to increase medication adherence in patients with hypertension and hyperglycemia and reduce complications from other chronic diseases by providing certain prescription medications for free. Medicines include Nifedipine Extended-Release Tablets, Metoprolol Tartrate Tablets, Captopril Tablets, Hydrochlorothiazide Tablets, Clonidine Hydrochloride Tablets for hypertension and Metformin Hydrochloride Extended-Release Tablets, Gliclazide Modified-Release Tablets for type 2 diabetes. For patients who can receive free drugs, the essentials are as follows: (1) having local household registrations; (2) enrolled in the Family Doctor Contracted Service. Subsequently, family physicians will develop personalized treatment plans, and then free medications can be obtained from the community health center. The details of the implementation of the program can be seen in the Supplementary Figure S1.

### Non-pharmaceutical intervention during the COVID-19 pandemic

In addition to free hypertension pharmaceutical intervention, non-pharmaceutical interventions (NPIs) exist concurrently during the COVID-19 pandemic in early 2020. Social distancing is a set of measures of NPIs, which has been recommended to reduce the chance of infection among high-risk populations and the burden on health care systems. It is useful for the containment and delay of contagious diseases by maintaining a physical distance between people and reducing the number of times people come into close contact with each other.

When the Chinese government officially confirmed on January 20, 2020 that the Novel Coronavirus can transmit from person to person, a social distancing policy rapidly swept across most cities in China. Impressively, within-city population mobility declines sharply and people chose to stay at home. As of March 14, 2020, when new confirmed cases decreased to 20, most provinces had lowered the level of emergency responses and within-city population movements gradually returned to pre-pandemic levels. We thus defined the strict social distancing period as January 20 to March 14. Stay-at-home made both physical activities and social support weakened during this period, which enabled us to examine the adverse effects on stroke mortality.

### Data collection

We retrospectively collected the routine surveillance data of stroke deaths reported by the Death Registration System of the Huzhou Municipal Center for Disease Prevention and Control from 1 January 2013, through 31 December 2020.

We obtained within-city mobility data from the Baidu Migration website,^1^ a map offered by a Chinese multinational technology company called Baidu. Specifically, Baidu Migration provides three migration intensity indices: the daily in-migration index (IMI) of a city, the daily out-migration index (OMI) of a city, and the daily within-city migration index (WCMI). And WCMI is the exponent result of the ratio of the daily number of people flowing in the city to the number of residents of the city, which can be used to measure the degree of recovery of city vitality. In this study, we only extracted the daily WCMI in 2019–2020 at the city-day level, which represents the population movements only in Huzhou itself per day.

Our research was conducted in accordance with the Declaration of Helsinki. No informed consents were required by patients, all data included in this study were kept confidential without personal identifiers.

### Statistical analysis

We classified the data on stroke deaths by calendar time and districts. The data on stroke deaths from 2013 to 2020 were then organized into weekly time series data. Considering the characteristic of Huzhou, inter-city population mobility can be deemed relatively small in our study, thus we only focused on the changes in within-city population movements, and compared the values of WCMI as Jan 20 to March 14 in 2020 with corresponding values from the same lunar calendar periods in 2019.

Descriptive statistics were presented by t-test, linear regression and correlation analysis. Moreover, Serfling regression model was applied to estimate the effects of pharmaceutical intervention and social distancing at the population level, respectively. Serfling model was initially designed to develop a “standard curve of expected seasonal mortality” (17). Weekly stroke deaths are characterized by time series data. After initial exploratory data analysis and model validation, we formulated an adjusted Serfling model using Fourier terms with a 52-weeks cycle and a 26-weeks cycle in this study. The time metric in the model was continuous calendar time in weeks unit, a variable that was included as a covariate to approximate the secular trend of stroke deaths, wherein sinusoidal transformations of the time metric were introduced in pairs to capture the seasonality of stroke deaths over time. To mitigate the risk of spurious results in our analysis, we also fitted Serfling regression models in other regions with similar characteristics with Deqing but without a free pharmacy program. A two-tailed *p-*value < 0.05 was considered statistically significant. All statistical analyses were conducted using R software, version 4.1.1 (R Foundation for Statistical Computing).

## Results

### Temporal trend of stroke mortality in Huzhou

The number of stroke deaths shows a declining trend over years from 2013 to 2020 in Deqing, Huzhou (*β* = −7.17, *p* = 0.29), whereas the other four districts or counties in Huzhou remain increasing (all *β* > 0), as shown in Supplementary Table S1.

### Impact of the free pharmaceutical intervention program

**Figure 1:**
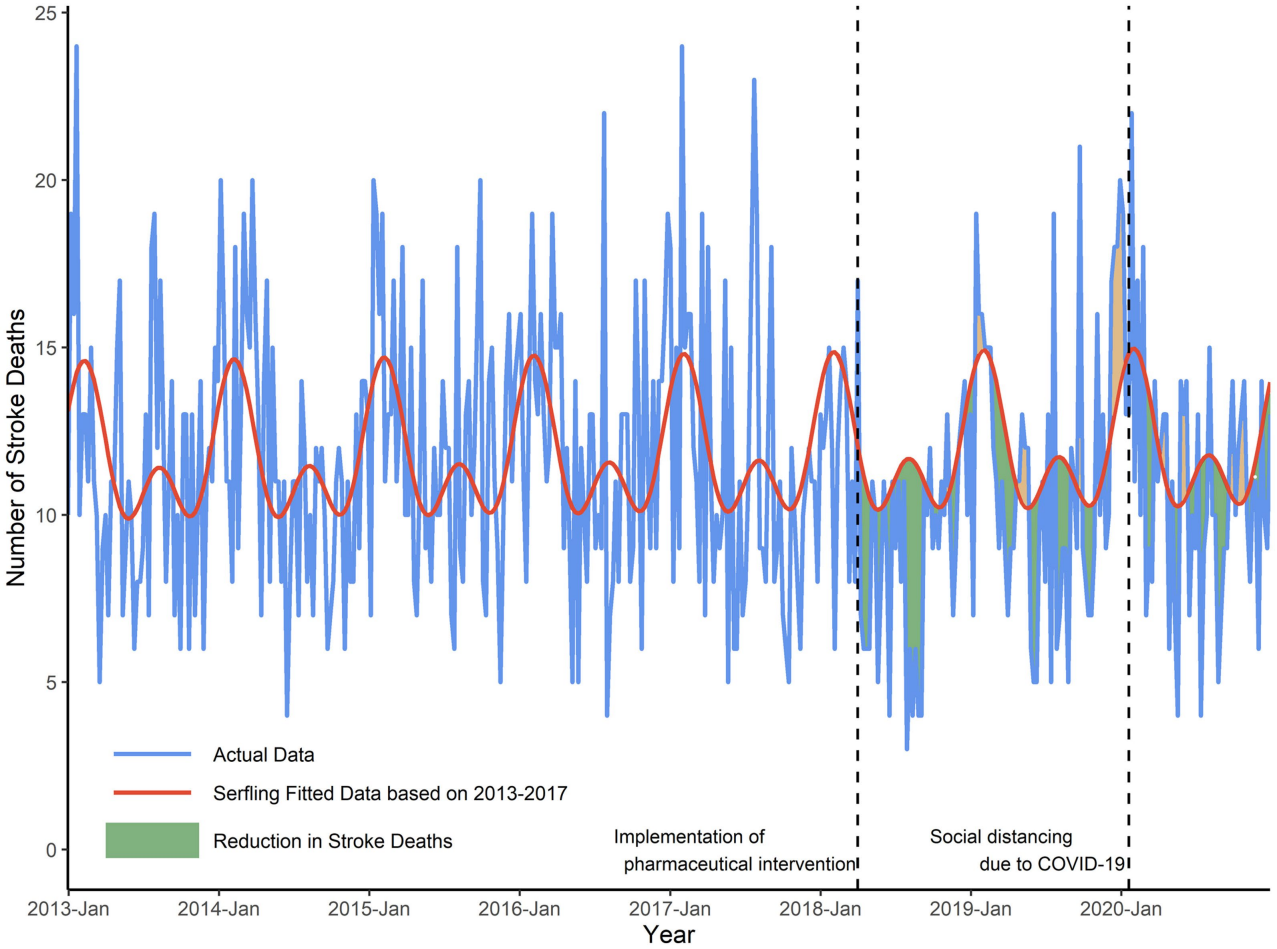
Number of weekly stroke deaths in Deqing, 2013–2020. Blue line depicted actual number of stroke deaths per week from January 2013 to December 2020. Red line predicted trends of stroke deaths using Serfling regression model based on 2013–2017 historic data in the counterfactual world in which pharmaceutical and non-pharmaceutical intervention were not implemented. Green shadows were presented as the reduction in stroke deaths, indicating those timepoints when actual number of stroke deaths was less than predicted number of deaths after the free hypertension pharmacy program. The dashed vertical lines showed the time when the free pharmaceutical intervention program in Deqing (April 1, 2018) and large-scale social distancing due to the pandemic of COVID-19 (January 20, 2020) were implemented.

The number of weekly stroke deaths in Deqing (blue line) was demonstrated with fitted data (red line) using a Serfling model (model estimates fitted on the data of 2013–2017 stroke deaths and predictions made on 2018–2020), as shown in Figure 1.

**Figure 2:**
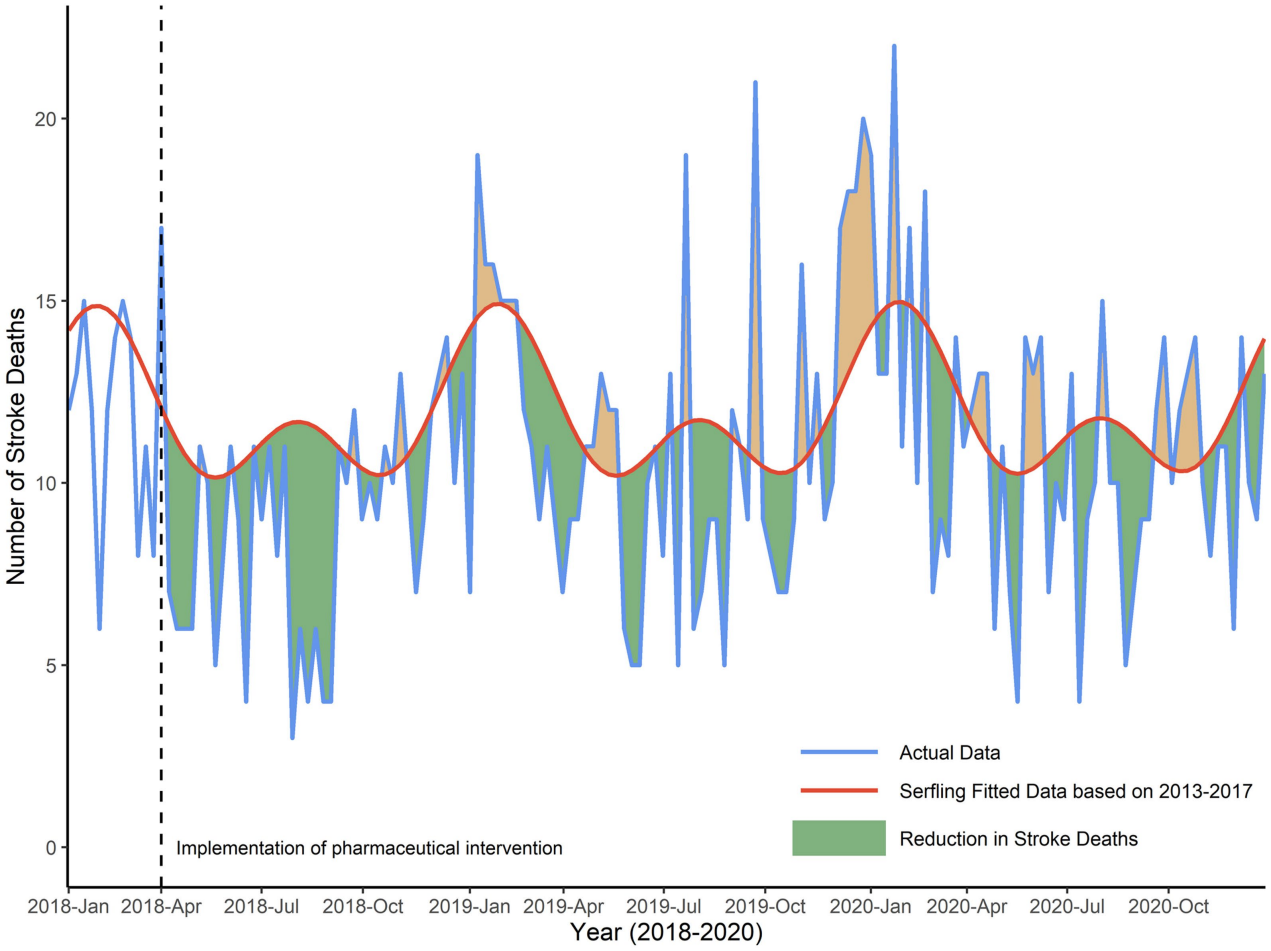
Reduction in number of weekly stroke deaths in Deqing, 2018–2020. Green shadows were presented as the reduction in actual stroke deaths compared with predicted numbers from 2018 to 2020, due to the presence of both pharmaceutical and non-pharmaceutical interventions. Orange shadows were presented as the increase in actual stroke deaths compared with predicted numbers.

The total number of reported deaths of stroke since the intervention program for the 2018–2020 season was 1,514, and 176 persons were deemed in reduction of expected for the cyclical regression. After the implementation of pharmaceutical intervention program since April 2018, as shown in Figure 2, the actual number of stroke deaths was significantly lower by 10% (95% CI, 6–15%; *p* < 0.001) from April 2018 to December 2020 in Deqing, compared to the predicted number. Under the assumption of a free hypertension program implemented in other four districts or counties in Huzhou, Serfling regression results showed that the reduction in actual stroke deaths compared with predicted numbers from 2018 to 2020 was only statistically significant in Anji county (*t* = −2.59, *p* < 0.01), as shown in Supplementary Table S2.

**Figure 3:**
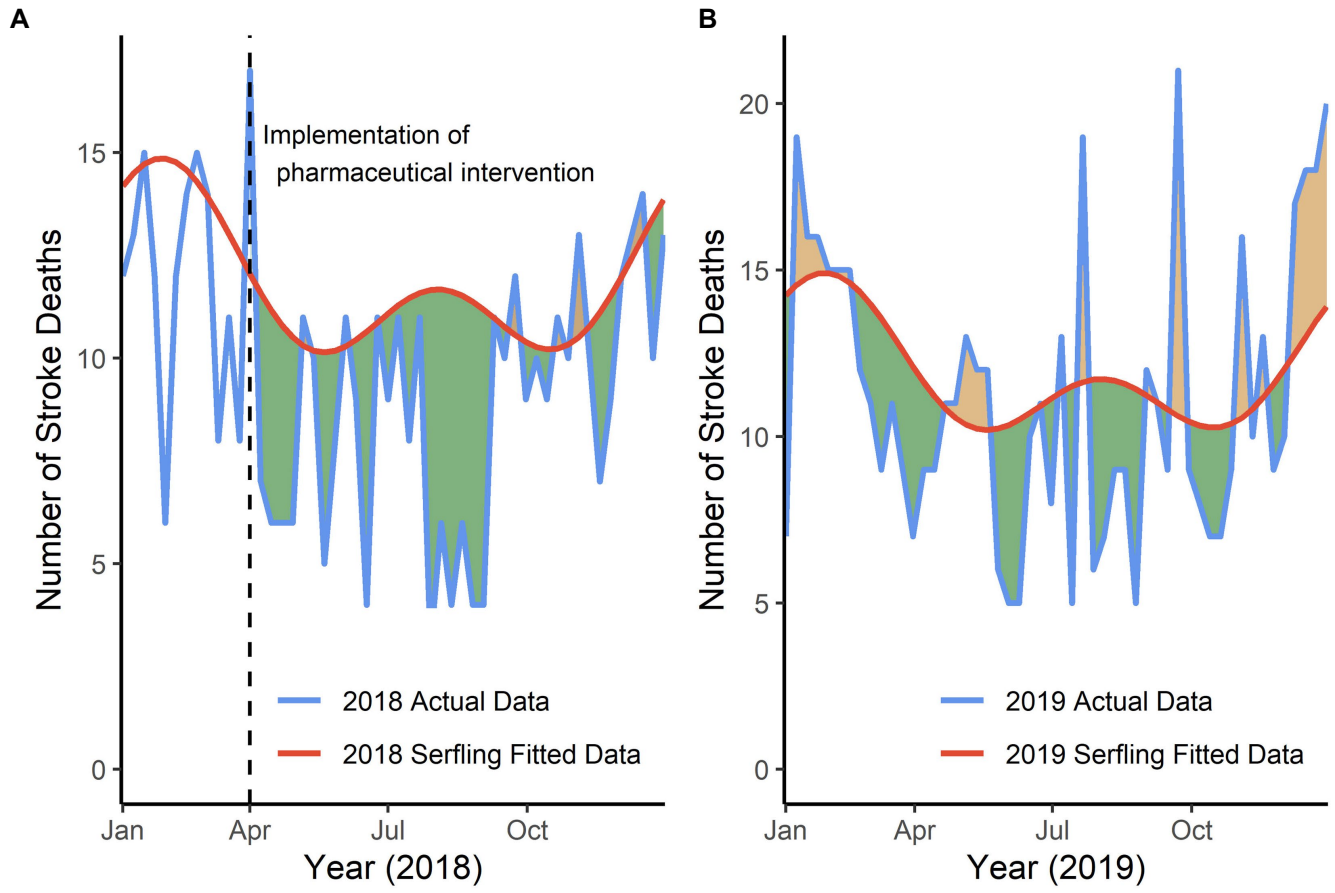
Reduction in number of weekly stroke deaths in Deqing, 2018–2019, respectively. **(A)** Green shadows were presented as the reduction in actual stroke deaths compared with predicted numbers since April 2018, in the presence of only pharmaceutical intervention effect, in which there was a significant reduction of 19% (*t* = −4.26, *p* < 0.001). **(B)** Green shadows were presented as the reduction in actual stroke deaths compared with predicted numbers in year 2019, in the presence of only pharmaceutical intervention effect, in which there was a reduction of 7% (*t* = −1.47, *p* = 0.15).

To be specific, as shown in the left panel of Figure 3, the actual number was significantly lower than the predicted number, with a reduction of 19% (95% CI, 10–28%; *p* < 0.001) in 2018. However, compared to the predicted number of stroke deaths, the reduction of stroke deaths was not statistically significant both in 2019 and 2020. As shown in the right panel of Figure 3, the actual number was lower by 7% (95% CI, −2 – 16%; *p* = 0.13) in 2019. And there was a reduction of 8% (95% CI, −0.4 – 16%; *p* = 0.06) in 2020 (figure not shown).

Besides, the proportion of weekly reduction in the number of stroke deaths showed no significant change over time in 2018 (*r* = −0.21, *p* = 0.19) and 2019 (*r* = −0.23, *p* = 0.10) in Deqing.

### Impact of social distancing during the COVID-19 pandemic

During the COVID-19 pandemic in early 2020, the average number of monthly stroke deaths was 51 in the first half of 2020, whereas it was 47 in the first half of 2019 in Deqing. And the average numbers of monthly stroke deaths from 2013 to 2017 were all greater than 50, the number in 2018 would not be included in the comparison due to the implementation of the intervention program in April.

Specifically, we calculated changes in population movements as January 20 to March 14 in 2020 and compared the findings with corresponding changes from the same lunar calendar periods in 2019, during which the city was free of COVID-19 incidence and social distancing, we observed a significant decrease in the daily WCMI in 2020 (95% CI, 1.05–1.64, *p* < 0.001).

**Figure 4:**
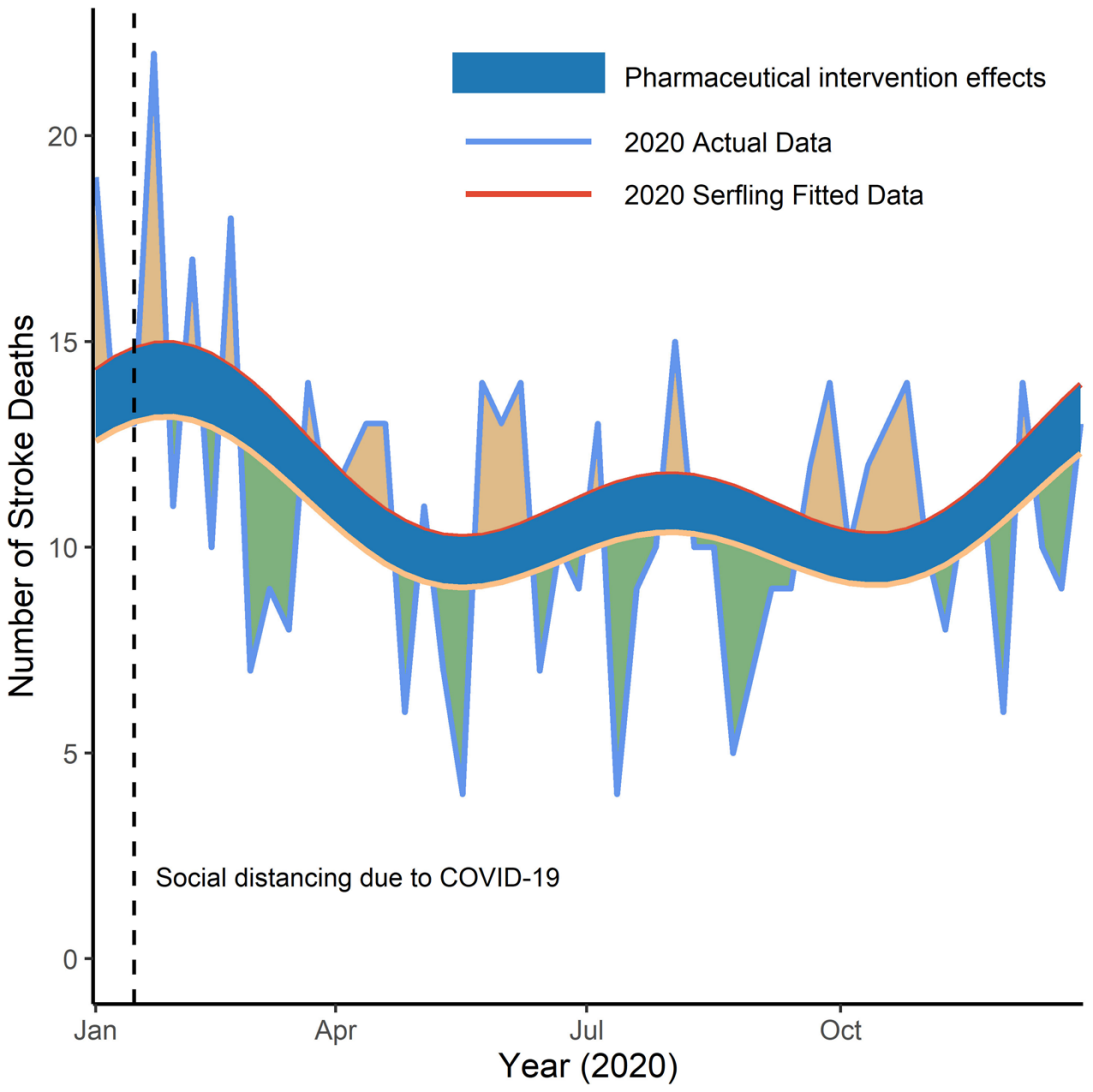
Reduction in number of weekly stroke deaths in Deqing, 2020. Yellow line represented adjusted predicted stroke deaths in 2020 after a large-scale social distancing due to the pandemic of COVID-19 was implemented. Blue shadows were presented as the pharmaceutical intervention effects throughout year 2020. Green shadows were presented as the reduction and orange shadows as the increase in actual stroke deaths compared with predicted numbers.

To further disentangle the social distancing effect from the pharmaceutical intervention effect, we assumed that the decrease in the number of deaths caused by the pharmaceutical intervention throughout the year 2020 in Deqing was identical to the average decreasing level from April 2018 to December 2019, which was approximately equal to 12% (95% CI, 6–18%; *p* < 0.001), and then quantified an additional increase caused by social distancing was 5% (95% CI, −4 – 14%; *p* = 0.28), as shown in Figure 4. Finally, there still existed a reduction of 8% in the number of weekly stroke deaths in 2020 compared with predicted data, though it is not statistically significant.

## Discussion

In this study, we found that free pharmaceutical intervention was effective on the control of blood pressure, and the mortality of stroke also decreased. And stroke mortality was estimated decreasing 10% since the implementation of the intervention program. Specifically, there was a reduction of 19% in 2018. We also found that in the counterfactual world in which the other four districts or counties in Huzhou implemented the free pharmacy program, Anji was the only region that had stroke deaths declined. The decline could be attributed to the implementation of an intervention project launched by Fuwai Hospital in China. This comprehensive intervention project aimed at reducing the morbidity, recurrence, and mortality of CVD, Anji had participated in the program since 2015. As a result, compared with other regions in Huzhou, we concluded there occurred a decrease in stroke mortality in Deqing with the implementation of the free pharmacy program.

Previous studies of high blood pressure and CVD mainly focused on two aspects: one is the association between blood pressure and risk of CVD across different blood pressure levels, and the other is the cost-effectiveness of hypertension treatment for CVD prevention, whereas no evidence has ever been presented for quantitative evaluation on the impact of the implementation of a free hypertension pharmacy program on CVD prevention. The findings from our study add to existing evidence on the benefits of antihypertensive drug therapy by quantifying the impact on stroke mortality. In our large study, we investigated the effectiveness and degree of pharmaceutical intervention in decreasing stroke mortality at the population level. Our findings are in line with those of the randomized controlled trials, findings from large-scale analyses of randomized trials suggested that antihypertensive medications can be viewed as an effective tool to prevent cardiovascular mortality and morbidity, rather than simply reducing blood pressure (5, 18). A detailed study of age-stratified and blood-pressure-stratified effect of antihypertensive medications found pharmacological blood pressure reduction to be effective for CVD prevention across a wide range of ages, with no significant difference in relative risk reductions among different baseline systolic or diastolic blood pressure levels. It suggested effectiveness for antihypertensive treatment for older adults, even when their blood pressure is not highly abnormal (19). A modeling study in China suggested that treating all hypertension would prevent about 690,000 strokes (20). On contrary, it is worth noting that some researchers still insisted on common clinical practice for antihypertensive treatment that lowering blood pressure would have an uncertain or even detrimental effect. For instance, the UK NICE guideline recommends blood pressure-lowering treatment for primary prevention of CVD is not considered relevant when baseline systolic blood pressure is less than 140 mm Hg (21). Likewise, New Zealand’s Ministry of Health recommends screening adults for overall cardiovascular risk at first, and only individuals diagnosed at high risk for CVD and together with high blood pressure can receive antihypertensive drug treatment (22).

Adherence to regular and adequate chronic medications is a barrier for low-and middle-income residents. To reduce the burden of medical costs and encourage access to medicines, full coverage policies have been established in many countries worldwide. Few studies, however, have examined the effectiveness of CVD mortality on patients with hypertension accessing free medications. The relationship between costs and medication adherence has been widely studied (23). For example, Brazilian ‘Farmácia Popular’ program offered 17 kinds of hypertensive and diabetic medicines for free and improved adherence remarkably (24). A field survey in rural communities in Shandong Province in China also suggested that the provision of basic medicines at no charge for hypertensive patients increased medication adherence and overall all, patients in the intervention group had their blood pressure controlled lower than before the intervention, which is consistent with the evidence from our study (25). Recently, a modeling study in China also implied that full implementation of the essential medicines program will be important for reaping the benefits of improved hypertension control for preventing CVD (20).

The second main finding of our study was the observation that an extra 5% increase in the mortality of stroke during the COVID-19 pandemic, which may be attributable to the social distancing policy. But it wasn’t statistically significant, part of the reason for this was the short observation period of the adverse effect of COVID-19. On the other hand, it is precisely because of the existence of pharmaceutical intervention effect that the impact of social distancing on the changes of stroke mortality was not significant. However, the large-scale social distancing did greatly decrease within-city mobility among people from all walks of life during the outbreak, possibly leading to a lack of physical activity among all age groups, especially older people. An analysis of more than 150,000 patients with baseline hypertension with an average follow-up of 7.1 years showed that, compared with patients in the lowest quartile of physical activity, other groups all had a significantly lower risk of death from stroke, regardless of exercise intensity (26). Even for those who do not meet the minimum recommended exercise intensity, the risk of cardiovascular death also decreased significantly (27, 28). Social distancing not only caused a direct reduction in physical activity but also had a negative effect on mental health during the pandemic. Multiple longitudinal studies showed that individuals who experienced social isolation and loneliness tended to have more unhealthy behaviors (29–31), such as smoking and alcohol abuse, that may further be associated with the progression of CVD. Evidence from a large US prospective cohort study suggested social isolation and loneliness in the era of COVID-19 were independently associated with a modestly higher risk of CVD (32). Results from a population-based cohort study in rural areas in China demonstrated that older people with depression have increased all-cause mortality, which was not confounded by other cardiovascular risk factors. These indicate the need for more psychological interventions for people with CVD or risk factors of CVD (33).

Social distancing policy can also have a negative effect on timely medical care help. One hypothesis explaining the phenomenon may be that patients were fear of infection or contagion during the outbreak. The feeling may have been amplified by stay-at-home orders due to the strict social distancing policy and overwhelming news about the virus from the media during early 2020. However, delays in seeking medical care or even not seeking care would have a detrimental effect on stroke outcomes. Results from a Hong Kong study showed that there was a prolongation in stroke onset to hospital arrival time and a significant reduction in individuals arriving at the hospital within 4.5 h during the COVID-19 pandemic (34). Several studies in other countries such as United States, Brazil, Spain reported similar findings (35–37). Also, as the therapeutic time window for reperfusion for acute ischemic stroke is narrow (38), any delays in treatment would harm patients’ quality of life, and increase the risk of recurrence and mortality. Further studies based on retrospective population-based cohorts are needed to investigate how COVID-19 has led to changes in stroke epidemiology and the long-term impact on its clinical outcomes. Moreover, studies focusing on the association between individual patients’ behavior during COVID-19 and their functional outcomes are warranted.

Possible mechanisms have been proposed to elucidate the association between blood pressure lowering and stroke prevention. Repeated fluctuations in blood flow caused by the rise or fall of blood pressure increase mechanical stress on the arterial walls, leading to the process of vascular remodeling, such as arterial stiffening (39, 40), endothelial dysfunction (41–43), platelet activation (44), and inflammation (43, 45, 46). The human brain consists of tens of billions of neurons, due to its structural complexity and interconnected neural circuit, cerebrovascular microvasculature is especially vulnerable to blood fluctuations, possibly leading to cerebral small vessel disease like stroke (47, 48).

The mechanisms through which social distancing is associated with stroke may involve both decreasing activities of daily living and changing health status. The status of social distancing may affect cardiovascular health by dysregulating neuroendocrine system, disturbing autonomic function (49), triggering inflammatory reactions (50), and increasing long-term allostatic load (51). Also, the experiences of isolation and loneliness may directly activate the hypothalamic–pituitary–adrenal axis and the sympathetic nervous system, leading to enhanced inflammation and oxidative stress, then developing atherosclerosis and elevated systolic blood pressure (52, 53).

The main strength of our study is that we formulated a regression model under counterfactual scenarios and quantify the effect caused by interventions. Firstly, compared with other intervention evaluation methods in time series design, Serfling regression model does a reasonable job approximating the secular trend of stroke deaths by treating cyclical calendar time as a continuous predictor, wherein sinusoidal transformations of the time metric are introduced in pairs to capture the patterns of seasonally varying outcomes over time. Secondly, our study covered long-time periods in settings with large repositories of historical data from healthcare administrations and the validity was high for evaluating the temporality between free hypertension pharmacy program and stroke mortality. Thirdly, our study investigated not only the impact of the free provision of essential medications to prevent stroke, but also the adverse effects of changes in lifestyle and mental health status on stroke mortality due to social distancing.

Several limitations should be noted. Firstly, lagged effects of medications in our model were not considered. But it had been proved that the mean of the finalized model residuals is close to zero and autocorrelation function plot suggests there is no significant correlation in the residual series. It may partially attenuate the estimates but would not change conclusions substantially. Secondly, no specific data were available on how many patients in the population had received free antihypertensive treatment in our study. But we know the number of patients receiving free medications was the highest in 2018, it decreased in 2019 and slightly increased in 2020, which kept in line with the decreasing trend in stroke mortality over time. We hope to make improvements and analyze the association between the number of patients receiving different classes of drugs and the changes in stroke mortality in future studies. Thirdly, we could not rule out the influence of some unmeasured confounders, for instance, the provision of free risk factor modifying medications may also have a positive effect on the prevention of other conditions, such as diabetes, and kidney diseases. Fourthly, the location of the intervention program is in a developed and rich county, which may slightly overestimate the impact and limit the generalizability of our findings.

Further research that bypass the abovementioned limitations can be carried out to advance the evidence on blood pressure lowering and stroke prevention. Low-cost essential antihypertensive medications for hypertension control have been proven cost-effective by many researchers, we hope to roll out intervention programs and conduct a more in-depth evaluation of the impact over time. What’s more, we can evaluate the intervention effect at the individual level in the future. In addition, a detailed subgroup analysis on changes in mortality by gender or age can also be included in the analysis.

## Conclusion

We quantified the impact of free pharmaceutical intervention and social distancing in changing stroke mortality in Deqing, Zhejiang Province. Notably, our study examined the degree of hypertension control that would minimize the risk of stroke mortality and it suggested free hypertension pharmacy program has great potential to prevent at least 10% of stroke deaths in our country. These findings highlight the effectiveness of promoting free medications for preventing stroke in patients with hypertension.

Free supply of low-cost, essential medications that target patients with hypertension at increased risk of stroke could be taken into account in formulating public health policies and guiding allocations of health care resources, which may be cost-effective in reducing disease burden and economic burden in the future.

## Data availability statement

The raw data supporting the conclusions of this article will be made available by the authors, without undue reservation.

## Ethics statement

Ethical review and approval was not required for the study on human participants in accordance with the local legislation and institutional requirements. Written informed consent for participation was not required for this study in accordance with the national legislation and the institutional requirements.

## Author contributions

QZ: data analysis and interpretation, graphic visualization, and drafting and revision of the manuscript. MY, MJ, and PZ: data collection and interpretation, and revision of the manuscript. GQ and YY: study concept and design, and revision of manuscript. All authors contributed to the article and approved the submitted version.

## Funding

This study was supported by a grant for clinical research from Shanghai Municipal Health Commission (20214Y0020) to YYao, a grant from Shanghai Municipal Nature Science Foundation (22ZR1414600) to YYao, a grant for the young leading talents from Shanghai Municipal Health Commission (2022YQ076) to YYao. The funders had no role in study design, data collection and analysis, decision to publish, or preparation of the manuscript.

## Conflict of interest

The authors declare that the research was conducted in the absence of any commercial or financial relationships that could be construed as a potential conflict of interest.

## Publisher’s note

All claims expressed in this article are solely those of the authors and do not necessarily represent those of their affiliated organizations, or those of the publisher, the editors and the reviewers. Any product that may be evaluated in this article, or claim that may be made by its manufacturer, is not guaranteed or endorsed by the publisher.

## Footnotes

^1^ https://qianxi.baidu.com/
