# Supplementary material for: Impact of free hypertension pharmacy program and social distancing policy on stroke: A longitudinal study
Source: Front Public Health. 2023 Apr 18;11:1142299. doi: 10.3389/fpubh.2023.1142299 (PMC10151749; doi:10.3389/fpubh.2023.1142299)
Supplement: Supplementary file 1 [file Table_1.DOCX]

***Supplementary Material***

**Impact of Free Hypertension Pharmacy Program and Social Distancing Policy on Stroke: A Longitudinal Study**

Qi Zhou^1,†^, BS, Meihua Yu^2,†^, BS, Meihua Jin^2,*^, BS, Peng Zhang^2^, MS, Guoyou Qin^1^, PhD, Ye Yao^1,*^, PhD

1. Department of Biostatistics, School of Public Health, and The Key Laboratory of Public Health Safety of Ministry of Education, Fudan University, Shanghai, China
2. Huzhou Center for Disease Control and Prevention, Huzhou, Zhejiang Province, China

† These authors contributed equally to this work as first author.

* Correspondence:

Ye Yao

[yyao@fudan.edu.cn](mailto:yyao@fudan.edu.cn)

Meihua Jin

[huzhoujmh6821@163.com](mailto:huzhoujmh6821@163.com)

Figure S1. The process of the delivery of the pharmaceutical intervention to the participants

**Prerequisites for participation in the intervention program:**

1) hypertensive patients who have registered permanent residence in Deqing County;

2) hypertensive patients who are successfully enrolled in the Family Doctor Contracted Service.

Patients with hypertension visit their doctors in the designated community health center which is allocated based on the current home address.

The patient signs an informed consent form, and the doctor will determine the specific treatment plan and prescribe antihypertensive drugs.

Is blood pressure controlled well?

The doctor prescribes according to the previously determined treatment plan and the patient continues to receive free medications.

The doctor adjusts the treatment plan and the patient receives another class of free antihypertensive medications.

Yes

No

Blood pressure is still not well controlled.

Yes

Referral to a higher level hospital for visits.

Table S1. Temporal trend of stroke mortality in Huzhou from 2013 to 2020

|  | 2013 | 2014 | 2015 | 2016 | 2017 | 2018 | 2019 | 2020 | *β* | t | *P* value |
| --- | --- | --- | --- | --- | --- | --- | --- | --- | --- | --- | --- |
| Wuxing District | 636 | 547 | 577 | 523 | 519 | 453 | 618 | 708 | 5.75 | 0.44 | 0.68 |
| Nanxun District | 571 | 602 | 577 | 627 | 667 | 551 | 742 | 690 | 17.79 | 2.14 | 0.08 |
| Deqing County | 594 | 601 | 633 | 632 | 591 | 502 | 580 | 585 | -7.17 | -1.17 | 0.29 |
| Changxing County | 499 | 496 | 485 | 642 | 513 | 566 | 639 | 627 | 20.54 | 2.63 | 0.04 |
| Anji County | 569 | 566 | 593 | 545 | 609 | 600 | 541 | 601 | 2.19 | 0.51 | 0.63 |

Table S2. Paired t-test results of the Serfling regression model in other four district/counties other than Deqing

|  | t-statistic | *P* value |
| --- | --- | --- |
| Wuxing District | t = 7.49 | *P* < 0.001 |
| Nanxun District | t = -1.72 | *P* = 0.09 |
| Changxing County | t = 0.66 | *P* = 0.51 |
| Anji County | t = -2.59 | *P* = 0.01 |
